# Experimental and Numerical Investigation on Fatigue Properties of Carbon Fiber Cross-Ply Laminates in Hygrothermal Environments

**DOI:** 10.3390/polym14091857

**Published:** 2022-04-30

**Authors:** Mingrui Xu, Benyin Zeng, Ziqian An, Xin Xiong, Xiaoquan Cheng

**Affiliations:** 1 School of Aeronautic Science and Engineering, Beihang University, Beijing 100083, China; laoben001@sina.com (B.Z.); anziqian@buaa.edu.cn (Z.A.); xiaoquan_cheng@buaa.edu.cn (X.C.); 2 Aviation Industry Corporation of China, Ltd. (AVIC), China Helicopter Research and Development Institute, Jingdezhen 333001, China; xiongx005@avic.com

**Keywords:** laminates, tensile fatigue, hygrothermal environments, fatigue properties, S–N curve

## Abstract

The fatigue properties of composite materials are degraded seriously in hygrothermal environments, so taking into account their influence is very important when evaluating the fatigue life of composite structures. Tensile fatigue experiments of carbon fiber reinforced resin composite cross-ply laminates were conducted in room temperature/dry (RTD), cool temperature/dry (CTD) and elevated temperature/wet (ETW) conditions. The S-N curves and fatigue failure modes of the cross-ply laminates were obtained in three conditions. On this basis, a finite element model was established to discuss the influence of temperature and moisture content on the fatigue properties, as well as a method for determining environmental factors of fatigue life of cross-ply laminates was established. The results show that the saturation moisture absorption and temperature have a significant influence on the tensile fatigue properties of cross-ply laminates. The high-cycle fatigue property is weakened significantly by the saturation moisture absorption and high temperature, but the low-cycle fatigue properties were strengthened in cool temperature conditions. The delamination failure mode in ETW is the most severe, presenting with an obvious necking phenomenon. The influence of temperature has a greater effect than that of moisture content, but moisture absorption would play its affect obviously when temperature exceeds 40 °C.

## 1. Introduction

Carbon fiber reinforced resin matrix composites (CFRP) have been widely used in aircraft structures due to their high specific strength and stiffness [1,2,3]. The composite structure of the aircraft not only needs to stand cyclic fatigue loads during the service period, but also may encounter harsh environmental conditions such as heat and humidity [4]. Due to them being sensitive to heat and humidity [5], an environment with elevated temperature and high moisture could seriously weaken the mechanical properties of the composite material structure, and that will make the fatigue problem more prominent. The durability of the composite structure in severe environment conditions has become a big challenge of their application in aerospace industries [6].

Some studies have been conducted on the fatigue properties of composite laminates in wet and thermal circumstances [7,8,9,10,11,12,13,14,15,16,17,18]. Mismatch in moisture expansion and thermal expansion coefficients between the fiber and matrix will induce wet stress and thermal stress, and then initiate micro-cracks and debonding in the interface of the fiber/matrix, reducing the structural bearing capacity [19,20]. Kawai et al. [21] investigated the effect of moisture absorption on the fatigue strength of plane fabric fiber quasi-isotropic composite laminates under constant stress amplitude and different stress ratios. The results indicate that the fatigue life of wet laminates is lower than that of dry laminates, and the fatigue strength of wet laminates is 11% lower than that of dry laminates at room temperature. Mcbagonluri et al. [22] compared and studied the tensile fatigue properties of vinyl glass fiber composites in a dry state, fresh water and salty water immersion. The S–N curve after moisture absorption is lower than that in the dry state, but the slope of the S–N curve in three environments is almost the same. Malpot et al. [23] investigated the effect of moisture absorption on the fatigue property of composites in three wet conditions (RH0, RH50% and RH100%) and found that when the cycle number was less than 10^4^, the fatigue life decreased with the increase in relative humidity. However, when the number of cycles is greater than 10^4^, the humidity condition hardly affects the fatigue life. Therefore, the effect of hygrothermal environment on high-cycle fatigue far exceeds that of low-cycle fatigue. Kawai et al. [24] investigated the fatigue property of three composites, AS4/PEEK, T800H/Polyimide and T800H/Epoxy, at high temperature (100 °C) with different lay-up angles. The AS4/PEEK composites were found to exhibit stronger fatigue strength except for the 0° lay-up, while the T800H/Polyimide composites had weaker fatigue strength. The fatigue strength of all the lay-up materials except for the 0° layup showed a significant decrease when the number of cycles reached 10^5^. In terms of numerical simulation, Shokrieh et al. [25,26] combined stress analysis, failure analysis and material performance attenuation to propose a fatigue model suitable for carbon fiber composite unidirectional bands. Harper et al. [27] established a FEM mode by using cohesive element to simulate fatigue delamination.

All the investigations above focus on the individual effect on the fatigue property of composites about temperature and moisture absorption, so there are few studies on the combined effects of moisture and temperature. That contributes to the insufficiency of experimental verification, a deficient depth of numerical simulation, as well as the lack of the in-depth discussion upon the coupling between moisture and temperature.

We have previously investigated the fatigue property of carbon fiber cross-ply laminates under hygrothermal environmental conditions in the article [28] using the method of experiments and numerical simulations, and then established an analysis model about the environmental influence factor of fatigue life for laminates. The effect of the hygrothermal environment on the properties of laminates largely depends on the influence of the matrix and the matrix/fiber interface. The sensitivity of angle-ply laminates to that under the hygrothermal environment is much stronger than that of cross-ply laminates. Therefore, we continue this investigation to gain a deeper understanding of coupling effect of the temperature and moisture absorption as well as better to improve the analysis model.

## 2. Specimens and Tests

### 2.1. Specimen Design and Manufacture

**Figure 1:**
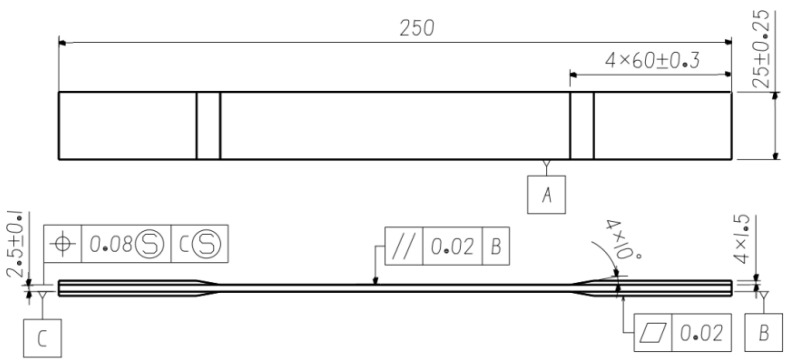
Static and tensile fatigue specimens.

In this work, T300 level CF3052/3238A carbon fiber-reinforced epoxy composite laminates were investigated whose configuration is shown in Figure 1. The specimens were designed based on ASTM D3479M [29] and a typical configuration of a [(45/−45)]_8_ lay-up was selected based on the application in rotorcraft industry. Prepregs were supplied by Guangwei Composites Co., Ltd. (Weihai, China). Laminates were manufactured by the AVIC composite corporation then. The maximum temperature for curing of laminates was 120 °C and they were kept for 120 mins under that condition. A pressure of 0.45 MPa was applied for the whole curing cycle.

The test category and number of specimens are presented in Table 1. Among them, there are 3 moisture absorption specimens, which were used to determine the saturated moisture absorption state and time of all specimens. There are three experiment environments, namely in the room temperature and dry condition (RTD), in the cool temperature and dry condition (CTD, −40 °C) as well as in the elevated temperature and wet condition (ETW, 55 °C). In order to determine the fatigue test load, the corresponding static tensile tests were conducted first.

### 2.2. Experimental Procedures

Three kinds of experiments were conducted, involving moisture absorption, tensile and tensile–tensile fatigue tests. All the specimens of ETW were firstly put in the hot water to reach wet condition, and then tensile and tensile–tensile fatigue tests were performed in RTD, RTW and ETW. When testing, the thermal condition was provided by the conditioning chamber.

#### 2.2.1. Moisture Absorption

Moisture absorption experiments were designed based on HB7401 [30] and ASTM Standard D5229 [31]. The specimens in ETW were immersed in a deionized water tank to achieve the moisture adsorption equilibrium as the water temperature was set to be 70 °C which is lower than the glass transmission of the matrix. Weights of specimens were regularly measured by an analytical balance with the accuracy of 0.1 mg. The specimens were cleaned by the absorbent cloth before weighing, and the time of weighing was within 5 min. Moisture equilibrium was reached when the moisture content met the following equation [31].

(1) $$M(\%)=\frac{W_{i}-W_{0}}{W_{0}}\times 100\%$$

where *M* is the moisture absorption content of the test specimens, *W*_0_ and *W_i_* are the mass of the test specimen before and after moisture absorption.

#### 2.2.2. Tensile Fatigue Tests

**Figure 2:**
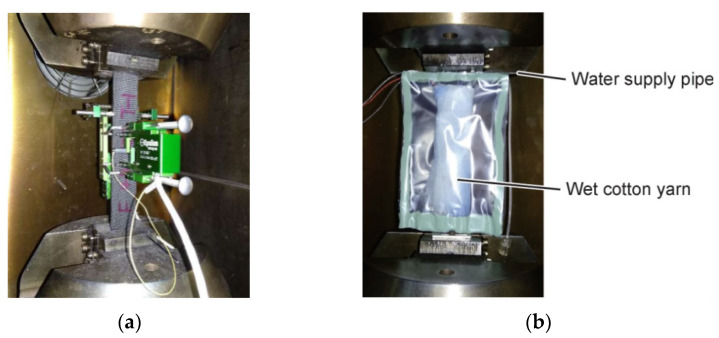
Test set for the tensile fatigue. (**a**) Test loading and measuring in RTD; (**b**) moisture retention of ETW test specimens.

As shown in Figure 2a, the fatigue tests were conducted on the Instron 8801 servo-hydraulic testing system (Instron Inc., Shanghai, China). Parameters of the sinusoidal waveform and stress ratio R = 0.0526 were set. RTD specimens were directly exposed to the environment and CTD/ETW specimens were placed in the conditioning chamber during testing. Hang extensometers with a maximum measurement range of 2.5 m in the longitudinal and transverse directions, respectively, were used to measure strains. The standard test method [29] points out that the loading frequency should ensure the change of the surface temperature of the test specimens does not exceed 10 °C. Therefore, an infrared temperature tester was used to monitor the temperature, and the loading frequency is set to 1 Hz according to the monitoring results. Due to the phenomenon of heating and water loss in the fatigue test of composite materials, the test specimens were moisturized by the method shown in Figure 2b under the ETW environment. Four stress levels which represent the maximum fatigue stress were selected based on some certain percentages of static strength. The test was terminated once the specimen was broken or the dynamic stiffness of the specimen was reduced by 10%.

## 3. Numerical Study

### 3.1. Basic Property Degradation

The wet and thermal environment seriously weaken the properties of the matrix and matrix/fiber interface, so its basic mechanical properties are deteriorated. The mechanical properties in RTD are shown in Table 2. The model established by Shan et al. [32] is used to describe the change law of basic mechanical properties in wet and thermal environments, in which the dimensionless T* considers the influence of temperature and moisture absorption on basic mechanical properties. T* can be described as

(2) $$T^{*}=\frac{T_{\mathrm{gw}}-T}{T_{g0}-T_{0}}$$

In which *T* is the current temperature, *T*_0_ is the starting temperature, *T*_gw_ is the glass transition temperature of resin when the current moisture absorption is *C*, and *T*_g0_ is the glass transition temperature of resin in dry state. *T*_gw_ and *T*_g0_ satisfy the following relationship,

(3) $$T_{\mathrm{gw}}=T_{g0}-gC$$

In which, *C* is the current moisture absorption of resin and g is the constant. The resin moisture absorption *C* is calculated from the moisture absorption content *M* of laminates, resin density $\rho_{m}$, laminate density ρ and resin volume content $V_{m}$ according to Equation (4),

(4) $$C=\frac{\rho M}{\rho_{m}V_{m}}$$

After the dimensionless T* is obtained, the strength and stiffness of laminates are attenuated according to Equation (5),

(5) $$\begin{array}{l} \frac{E_{11}}{E_{11}^{0}}=\frac{E_{22}}{E_{22}^{0}}={\left(T^{*}\right)}^{a}\\ \frac{X_{T}}{X_{T}^{0}}=\frac{Y_{T}}{Y_{T}^{0}}={\left(T^{*}\right)}^{b}\\ \frac{G_{12}}{G_{12}^{0}}={\left(T^{*}\right)}^{c}\\ \frac{S_{12}}{S_{12}^{0}}={\left(T^{*}\right)}^{d} \end{array}$$

In which, *E*_11_ and *E*_22_ are the elastic modulus of laminates along longitudinal and transverse directions under the current environment, respectively, $E_{11}^{0}$ and $E_{22}^{0}$ are elastic modulus along two directions in RTD, respectively, *X*_T_ and *Y*_T_ are the tensile strength along two directions under the current environment, respectively, $X_{T}^{0}$ and $Y_{T}^{0}$ are the tensile strength along two directions in RTD, respectively, *G*_12_ and *S*_12_ are the in-plane shear modulus and strength of laminates under the current environment, respectively, $G_{12}^{0}$ and $S_{12}^{0}$ are the in-plane shear modulus and strength in RTD, respectively. Finally, a, b, c, and d are the degradation constants of material properties in wet and thermal environments.

The parameters used in Equations (2)–(5) are shown in Table 2. Based on the test data in RTD, the calculated value of material properties in CTD and ETW are shown in Table 3. Compared with the simulation results, it was found that the theoretical value is in good agreement with the test value, and the correlation coefficients R^2^ is greater than 0.99, indicating that the prediction of mechanical property parameters for laminates is reliable.

### 3.2. Fatigue Failure Criterion and Property Degradation Model

In the process of fatigue loading, the residual strength and stiffness will show a downward trend. The degradation law is described as follows [25,26],

(6) $$\begin{array}{l} \frac{X_{T}\left(n,\sigma ,R\right)}{X_{T}}=\frac{X_{C}\left(n,\sigma ,R\right)}{X_{C}}=\frac{Y_{T}\left(n,\sigma ,R\right)}{Y_{T}}=\frac{Y_{C}\left(n,\sigma ,R\right)}{X_{C}}=1-\left[1-\left(\frac{\sigma_{1}}{X_{T}}\right)\right]{\left(\frac{n}{N}\right)}^{1.3218}\\ \frac{E_{11}\left(n,\sigma ,R\right)}{E_{11}}=1-\left[1-{\left(\frac{\sigma_{1}}{0.8857X_{T}}\right)}^{1/1.0702}\right]{\left(\frac{n}{N}\right)}^{0.5418}\\ \frac{S_{12}\left(n,\sigma ,R\right)}{S_{12}}=1-\left[1-\left(\frac{\tau_{12}}{S_{12}}\right)\right]{\left(\frac{n}{N}\right)}^{9.3459}\\ \frac{G_{12}\left(n,\sigma ,R\right)}{G_{12}}=1-\left[1-{\left(\frac{\tau_{12}}{4.7853S_{12}}\right)}^{1/14.9393}\right]{\left(\frac{n}{N}\right)}^{0.2623} \end{array}$$

where *X*_T_(*n*,*σ*,*R*), *X*_C_(*n*,*σ*,*R*), *Y*_T_(*n*,*σ*,*R*), *Y*_C_(*n*,*σ*,*R*) denote the residual strength along the transverse and longitudinal direction, respectively, as well as the subscript T and C is for tensile and compression. *E*_11_(*n*,*σ*,*R*), *S*_12_(*n*,*σ*,*R*), *G*_12_(*n*,*σ*,*R*) are the residual elastic modulus, in-plane shear strength and in-plane shear modulus respectively after n cycles, *X*_T_, *X*_C_, *Y*_T_, *Y*_C_, *E*_11_, *S*_12_, *G*_1_ are the corresponding strength and modulus, respectively, before fatigue testing, *σ*_1_ and *τ*_12_ are the normal stress and in-plane shear stress, respectively, and *N* is the fatigue life.

Damage criteria of composites were selected according to Shokrieh [25,26] (Equations (7)–(11)).

1. Longitudinal fiber tensile failure:
  (7) $$f_{1t}^{2}={\left(\frac{\sigma_{1}}{X_{T}\left(n,\sigma ,R\right)}\right)}^{2}+{\left(\frac{\tau_{12}}{S_{12}\left(n,\sigma ,R\right)}\right)}^{2}+{\left(\frac{\tau_{13}}{S_{13}\left(n,\sigma ,R\right)}\right)}^{2}\geq 1$$
2. Longitudinal fiber compressive failure:
  (8) $$f_{1c}^{2}=\left(\frac{\sigma_{1}}{X_{C}\left(n,\sigma ,R\right)}\right)\geq 1$$
3. Transverse fiber tensile failure:
  (9) $$f_{2t}^{2}={\left(\frac{\sigma_{2}}{Y_{T}\left(n,\sigma ,R\right)}\right)}^{2}+{\left(\frac{\tau_{12}}{S_{12}\left(n,\sigma ,R\right)}\right)}^{2}+{\left(\frac{\tau_{23}}{S_{23}\left(n,\sigma ,R\right)}\right)}^{2}\geq 1$$
4. Transverse fiber compressive failure:
  (10) $$f_{2c}^{2}=\left(\frac{\sigma_{2}}{Y_{C}\left(n,\sigma ,R\right)}\right)\geq 1$$
5. Fiber-matrix shear failure:
  (11) $$f_{12}^{2}={\left(\frac{\sigma_{1}}{X_{C}\left(n,\sigma ,R\right)}\right)}^{2}+{\left(\frac{\tau_{12}}{S_{12}\left(n,\sigma ,R\right)}\right)}^{2}+{\left(\frac{\tau_{23}}{S_{23}\left(n,\sigma ,R\right)}\right)}^{2}\geq 1$$
  where $\sigma_{i}$ and $\tau_{\mathrm{ij}}$ are the components of normal and shear stress. $S_{13}\left(n,\sigma ,R\right)$ and $S_{23}\left(n,\sigma ,R\right)$ are the ris1ual shear strength in the 1-3 and 2-3 planes after n cycles.

The stress–strain relationship of a single-layer lamina can be given as,

(12) $$\left[\begin{array}{c} \sigma_{1}\\ \sigma_{2}\\ \sigma_{3}\\ \begin{array}{l} \tau_{23}\\ \tau_{31}\\ \tau_{12} \end{array} \end{array}\right]=\left[\begin{array}{cccccc} C_{11} & C_{12} & C_{13} & 0 & 0 & 0\\ C_{21} & C_{22} & C_{23} & 0 & 0 & 0\\ C_{31} & C_{32} & C_{33} & 0 & 0 & 0\\ 0 & 0 & 0 & C_{44} & 0 & 0\\ 0 & 0 & 0 & 0 & C_{55} & 0\\ 0 & 0 & 0 & 0 & 0 & C_{66} \end{array}\right]\left[\begin{array}{l} \varepsilon_{1}\\ \varepsilon_{2}\\ \varepsilon_{3}\\ \gamma_{23}\\ \gamma_{31}\\ \gamma_{12} \end{array}\right]\;or\;\left\{\sigma \right\}=\left[C\right]\left\{\varepsilon \right\}$$

where $\varepsilon_{i}$ and $\gamma_{ij}$ represent linear and shear strain, respectively, and [C] denotes the three-dimensional stiffness matrix.

The damage quantity *D* is introduced to characterize the damage degree of the material. *D* = 0 means that the material is not damaged, and d = 1 means that the material is completely damaged. When damage occurs, the propagation was described by stiffness degradation as,

(13) $$\left\{\sigma \right\}=\left[\hat{C}\right]\left\{\varepsilon \right\}$$

where $\left[\hat{C}\right]$ is the reduced stiffness matrix, ${\hat{C}}_{ij}$ is given by, $\hat{C_{12}}=\left({1-d}_{1t}\right)\left({1-d}_{1c}\right)\left({1-d}_{2t}\right)\left({1-d}_{2c}\right)C_{12}$, $\hat{C_{12}}=\left(1-d_{1t}\right)\left(1-d_{1c}\right)\left(1-d_{2t}\right)\left(1-d_{2c}\right)C_{12}$, $\hat{C_{22}}=\left({1-d}_{2t}\right)\left({1-d}_{2c}\right)C_{22}$, $\hat{C_{13}}=\left(1-d_{1t}\right)\left(1-d_{1c}\right)C_{13}$, $\hat{C_{23}}=\left(1-d_{2t}\right)\left(1-d_{2c}\right)C_{23}$, $\hat{C_{33}}{=C}_{33}$, $\hat{C_{44}}=\left({1-d}_{12}\right)C_{44}$, $\hat{C_{55}}{=C}_{55}$, and $\hat{C_{66}}{=C}_{66}$. $d_{it}$ and $d_{ic}$ (*i* = 1, 2), respectively, express the damage degree of tensile and compression along 2 directions, and $d_{12}$ is for the change of in-plane shear damage. The damage variable $d_{a}$ is a continuous function of $f_{a}$ (*a* = 1t, 1c, 2t, 2c, 12),

(14) $$\begin{array}{l} d_{1t}=1-\frac{1}{f_{1t}}e^{\left(1-f_{1t})(\frac{X_{t}\varepsilon_{t}^{x}}{G_{1t}}\right)}=1-\frac{1}{f_{1t}}e^{\left(1-f_{1t})(\frac{X_{t}\varepsilon_{t}^{x}L_{c}}{W_{1t}}\right)}\\ d_{1c}=1-\frac{1}{f_{1c}}e^{\left(1-f_{1c})(\frac{X_{c}\varepsilon_{c}^{x}}{G_{1c}}\right)}=1-\frac{1}{f_{1c}}e^{\left(1-f_{1c})(\frac{X_{c}\varepsilon_{c}^{x}L_{c}}{W_{1c}}\right)}\\ d_{2t}=1-\frac{1}{f_{2t}}e^{\left(1-f_{2t})(\frac{Y_{t}\varepsilon_{t}^{y}}{G_{2t}}\right)}=1-\frac{1}{f_{2t}}e^{\left(1-f_{2t})(\frac{Y_{t}\varepsilon_{t}^{y}L_{c}}{W_{2t}}\right)}\\ d_{2c}=1-\frac{1}{f_{2c}}e^{\left(1-f_{2c})(\frac{Y_{c}\varepsilon_{c}^{y}}{G_{2c}}\right)}=1-\frac{1}{f_{2c}}e^{\left(1-f_{2c})(\frac{Y_{c}\varepsilon_{c}^{y}L_{c}}{W_{2c}}\right)}\\ d_{12}=1-\frac{\gamma (\gamma^{f}-\varepsilon_{12})}{\varepsilon_{12}(\gamma^{f}-\gamma )}\begin{array}{cc} & \left(\gamma^{f}=\frac{2G_{S}}{S_{12}}\right) \end{array} \end{array}$$

where $G_{a}$ (a = 1t, 1c, 2t, 2c, 12) is the fracture energy density of materials under different damage forms.

### 3.3. Fatigue Finite Element Model

**Figure 3:**
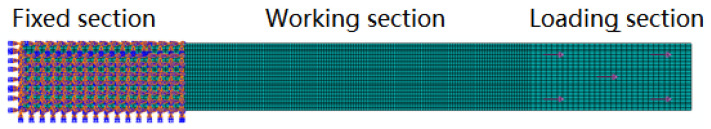
Finite element model.

As shown in Figure 3, Abaqus 6.14 is used to establish the finite element analysis model (FEA). The model is divided into three parts: a fixed support section, working section and loading section and laminates contains eight layers along the thickness direction. In order to improve the calculation accuracy, the grid element type is set to C3D8R, which contributes to 39,820 units in total. The load and constraint conditions are as follows: the load is applied through the in-plane shear force on the upper and lower surfaces of the loading section; translational constraints are applied to all nodes on the upper and lower surfaces of the fixed support section. The material property parameters in the FEA model are as shown in Table 4.

**Figure 4:**
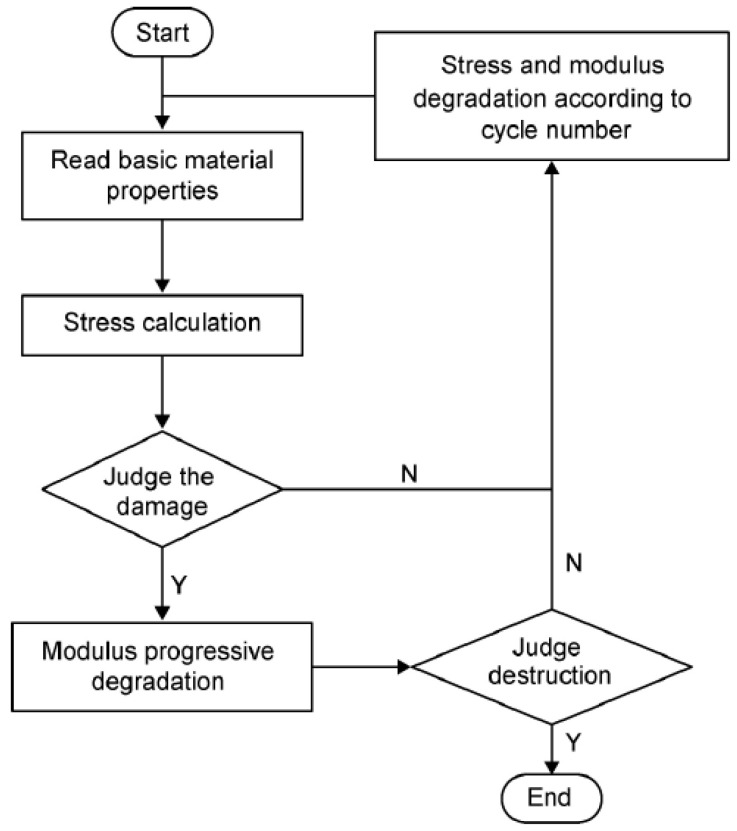
Flow chart of fatigue simulation.

A UMAT subroutine written in the Fortran language according to fatigue failure criterion and properties degradation model was used to carry out the progressive damage evolution of laminates, the flow chart of which is shown in Figure 4.

## 4. Results and Discussion

### 4.1. Tensile Tests

The average tensile properties in three environments are shown in Table 5. It can be found that, compared with the elastic modulus and tensile strength in RTD, they increase by 12.67% and 12.84%, respectively, in CTD, and decrease by −8.54% and −37.11%, respectively, in ETW. Therefore, it can draw a conclusion that the increase in matrix brittleness at cool temperature increases the elastic modulus and tensile strength correspondingly, while the coupling effect of high temperature and high moisture seriously weakens them.

### 4.2. Fatigue Test

#### 4.2.1. Hygrothermal Environment on the Fatigue Properties

The fatigue test results in three environments are shown in Table 6. A total of 16 specimens were conducted in each environment, which are divided into four groups according to the stress level, with an average of four in each group. The life is listed in the column of fatigue life and the stress level refers to the relative value of the average tensile strength.

The S–N curve, also known as the Wöhler curve, is obtained after a number of fatigue tests at different stress levels. In this paper, the two-parameter linear mode is applied [33],

(15) S=A+B*lgN

where A and B are material constants, S is max fatigue stress, N is the cycle number when composites are fractured.

**Figure 5:**
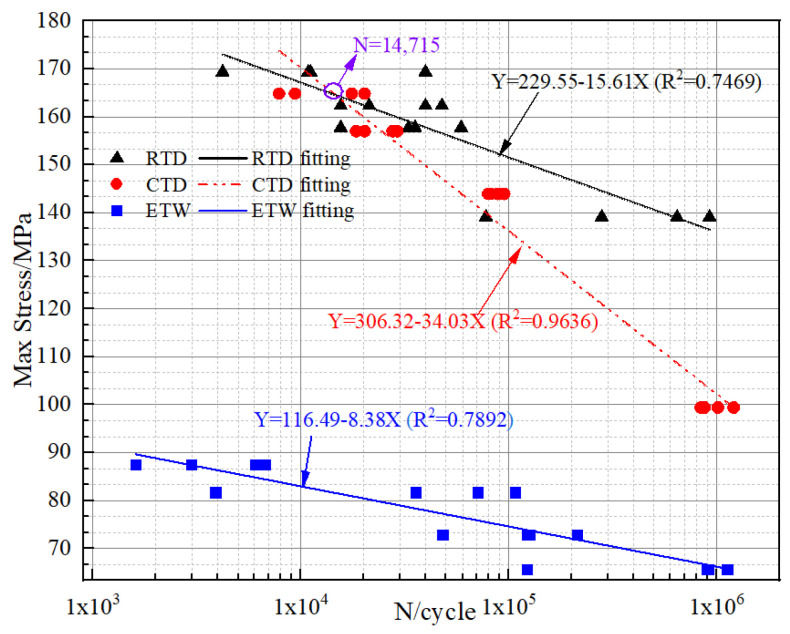
S-N curve of angel-ply laminates in three environments.

As shown in Figure 5, the fatigue S-N curves in RTD, CTD and ETW are fitted as Equations (16)–(18), respectively,

(16) S=229.55−15.61lgN

(17) S=306.32−34.03lgN

(18) S=116.49−8.38lgN

Figure 5 shows that, for the same cycle times, when the number of cycles n is greater than 14,715, the fatigue strength sequence from large to small is RTD, CTD, and ETW. When the number is less than 14,715, that sequence is CTD, RTD, and ETW. It means the cool temperature makes the high-cycle fatigue strength decrease, but it is beneficial to the low-cycle fatigue. In addition, the fatigue dispersion in RTD and ETW is comparable, and it is obviously reduced in CTD, which should be related to the embrittlement of the matrix at a cool temperature.

The decreasing rate of fatigue strength from large to small is RTD > ETW > CTD which means that the fatigue strength decreases most rapidly at cool temperatures. Taking the fitting curve results when the fatigue life is 10^6^ times for comparison, the fatigue strength in RTD, CTD and ETW are 135.9 MPa, 102.1 MPa and 66.2 MPa, respectively. Compared with in RTD, the fatigue strength in CTD and ETW is reduced by 24.88% and 51.28%, respectively. The coupling effect of high temperature and moisture on fatigue strength is significantly stronger than that of cool temperature alone.

#### 4.2.2. Failure Mode Analysis

**Figure 6:**
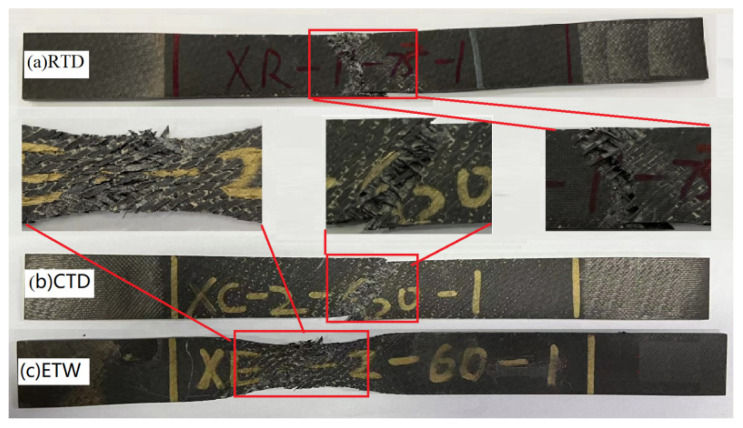
Fatigue failure morphology in three environments. (**a**) RTD; (**b**) CTD; (**c**) ETW.

**Figure 7:**
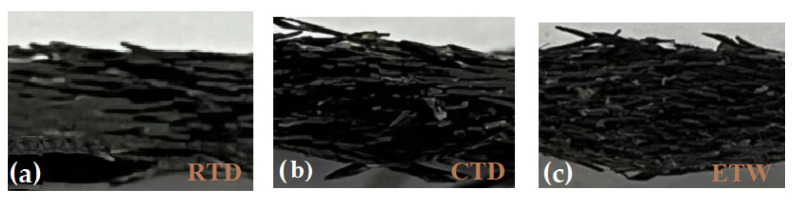
Fatigue failure morphology along the direction of thickness in three environments. (**a**) RTD; (**b**) CTD; (**c**) ETW.

The fatigue failure morphology in three environments is shown in Figure 6 and Figure 7. From Figure 6a and Figure 7a, it can be found that the fatigue failure is mainly matrix cracking and fiber fracture in RTD, accompanied by a certain 45° delamination propagation failure. At the same time, it can be seen from Figure 6b, Figure 7b,c and Figure 7b,c that failure modes such as fiber fracture, matrix cracking and delamination can be found in CTD and ETW as well. Delamination runs through the whole thickness direction and basically expands along the 0° direction in CTD and ETW. In addition, an obvious necking phenomenon can be seen in ETW which means the properties of matrix were weakened seriously. At last, it can be seen from Figure 7 that the delamination failure of ETW is the most severe in three environments.

### 4.3. Finite Element Results

#### 4.3.1. Model Verification

Table 7 presents the comparison of the fatigue life between the predicted values and the experimental values in three environments to verify the validity of the FEM model. It can be found that there is a larger error in the low-cycle section. However, in the high-cycle section, the simulation results are in good agreement with the experimental results, and the error is 13.2% at most. In general, the logarithmic life error is relatively small, which is less than 7%.

**Figure 8:**
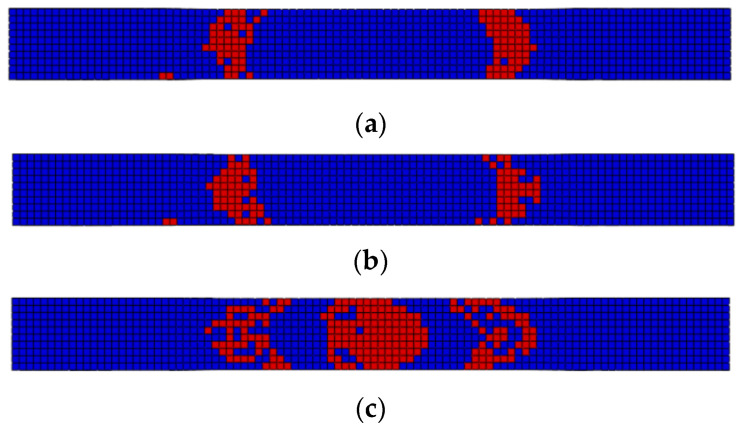
The final fatigue failure morphology calculated by the finite element method. (**a**) RTD; (**b**) CTD; (**c**) ETW.

The failure morphology obtained by finite element calculation is shown in Figure 8. From Figure 8a,b, it can be found that the fiber fracture damage occurs in a small area on the left and right of the specimen in RTD and CTD, and the damage distribution direction is at a certain angle with the edge of the specimen. From Figure 8c, it can be found that the specimen in ETW will have fiber failure in a wider range, which is similar to the failure mode shown in the test.

In conclusion, the model can effectively calculate the fatigue life of specimens in different environments and reflect their failure modes.

#### 4.3.2. Failure Process Analysis

According to the test results, the final fatigue failure of the specimen is mainly fiber fracture. Therefore, the development process of fiber fracture damage is observed with the help of the finite element model, so as to analyze the fatigue damage mechanism of the specimen.

**Figure 9:**
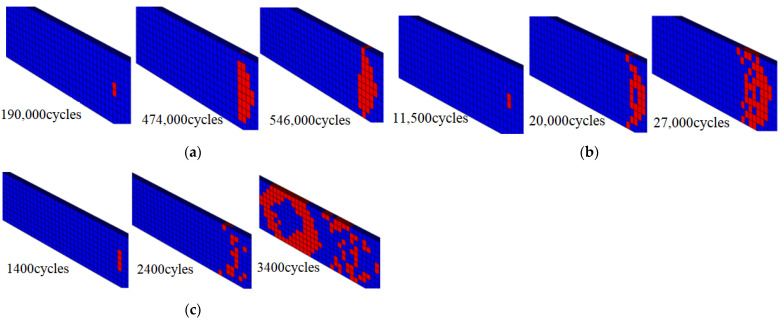
Fatigue failure process calculated by the finite element method. (**a**) RTD; (**b**) CTD; (**c**) ETW.

The damage evolution process of fiber fracture in three environments is shown in Figure 9 with the stress being set to 60% of their respective static strength. From Figure 9a in RTD, after 190,000 cycles of loading there is a small area of damage in the middle of the specimen, and then the damage extends roughly along the 45° direction of the edge of the specimen. After 474,000 cycles, the damage extends from the inside to the edge, and finally to the whole thickness and width of the specimen. From Figure 9b in CTD, the results of the fatigue failure process of the specimen are similar to those in RTD. from Figure 9c in ETW, after the initial damage occurs the damage propagation range is relatively larger, and finally a large area of fiber fracture occurs in the middle of the specimen then the overall life decreases significantly.

#### 4.3.3. Effect of Different Environments on Fatigue Life

In order to explore the coupling effect of temperature and moisture on the fatigue life of angel-ply laminates, the RTD environmental fatigue life of 10^6^ was taken as a fixed point, and the load at this point was kept unchanged at 135.5 MPa. The fatigue life of laminates at different temperatures (−40 °C, −20 °C, 0 °C, 20 °C, 40 °C and 55 °C) and different moisture absorption (0%, 0.5%, 1%, 1.5% and 1.8%) was calculated by the finite element model.

**Figure 10:**
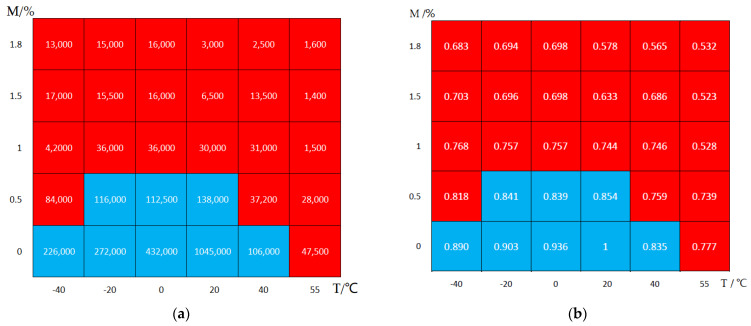
Fatigue life under different hygrothermal conditions. (**a**) Fatigue life; (**b**) Normalization of logarithmic life.

Figure 10 shows the fatigue life obtained by finite element calculation for different temperature and moisture combinations. Figure 10a is for fatigue life, and Figure 10b is the normalization type of logarithmic life. Taking the moisture absorption as the horizontal axis and the temperature as the vertical axis, the calculated values under different temperature and moisture absorption are drawn in a palace diagram. Taking the number of life cycles down to one tenth of the value at room temperature in dry state as the critical value, the effect of the hygrothermal environment on fatigue life is divided into a strong influence area and weak influence area, marked with red and blue, respectively. From the calculation results, when the moisture absorption content reaches 1.0% or the temperature exceeds 40 °C, all results are in the strong influence area.

Assuming that the effects of temperature and moisture are independent of each other, by fitting the above data, the environmental factors of fatigue life can be expressed as following,

(19) $$F=\frac{\log N}{\log N_{0}}=\left\{ \begin{array}{l} 1-0.358{\left(\frac{M}{M_{\infty }}\right)}^{0.725}-0.171{\left(\frac{T-T_{0}}{T_{1}-T_{0}}\right)}^{1.539}T\geq 20\;{}^{\circ}C\\ 1-0.230{\left(\frac{M}{M_{\infty }}\right)}^{0.771}-0.088{\left(\frac{T-T_{0}}{T_{1}-T_{0}}\right)}^{0.138}T<20\;{}^{\circ}C \end{array}\right.$$

where *F* is the environmental factors of fatigue life, *N* and *N*_0_ are the fatigue life in hygrothermal environment and in RTD, respectively, and *M* and *M*_∞_ are the current and maximum moisture absorption content, respectively. *T* is the current temperature, *T*_0_ is the reference temperature, and this formula takes 20 °C. *T*_1_ is the maximum temperature and 55 °C is taken in this formula.

## 5. Conclusions

In this paper, the tests of moisture absorption, static tensile and tensile–tensile fatigue were conducted on carbon fiber CF3250/3238A angle-ply laminates. Fatigue S–N curves and failure damage modes in three environments were obtained. Based on the experimental study, the finite element analysis model of progressive damage was established, and the influence of temperature and humidity on fatigue performance was discussed. Finally, the determination method of the environmental factors of fatigue life was established. Some conclusions were drawn as follows:

1. Compared with the RTD environment, the tensile strength in CTD and ETW increased by 12.84% and decreased by −37.11%, and the tensile modulus increased by 12.67% and decreased by −8.54%, respectively.
2. Only cool temperatures have obvious negative effects on the fatigue life dispersion of the test specimens, and high temperature and high moisture have no effect on that.
3. The decline rate of the S–N curve is the largest in CTD and the smallest in ETW. Cool temperature has a positive effect on low-cycle fatigue but has a negative effect on high-cycle fatigue.
4. When N = 10^6^, the fatigue limit in CTD and ETW is, respectively, decreased by 24.88% and 51.28% of that in RTD. The temperature plays a large role on the fatigue limit and the combination of high temperature and moisture has a much larger effect on that.
5. The failure morphology in three environments is similar, including fiber fracture, matrix cracking and delamination damage. However, the severity of failure is different, the failure of specimens in ETW is the most significant as well as the least in CTD. The fatigue morphology in ETW shows the obvious necking phenomenon since the matrix was seriously weakened.
6. Numerical analysis shows that the effect of temperature on fatigue property is significantly stronger than that of moisture absorption, and when the temperature exceeds 40 °C, the effects of moisture absorption are great.
7. A method for determining the environmental factors of fatigue life of angle-ply composite materials is proposed, which can be used for the life prediction of that in different environmental conditions.

## Figures and Tables

**Table 1:** Test types and specimen number.

Test Category	RTD	CTD	ETW
Moisture absorption	3		
Static tensile	3	3	3
Tension fatigue	16	16	16

**Table 2:** Material parameters [28].

$E_{11}^{0}/\mathrm{GPa}$	$E_{22}^{0}/\mathrm{GPa}$	$X_{T}^{0}/\mathrm{MPa}$	$Y_{T}^{0}/\mathrm{MPa}$	$G_{12}^{0}/\mathrm{GPa}$	$S_{12}^{0}/\mathrm{MPa}$	ρ	$\rho_{m}/g/{\mathrm{cm}}^{3}$	$V_{m}/\%$
54.3	54.3	680	680	3.26	116	1.42	1.23	45

**Table 3:** Empirical constants [28].

*T*_g0_/°C	*T*_0_/°C	*g/*°C/c	a	b	c	d
120	20	5	0.05	0.15	0.22	0.56

**Table 4:** Elastic engineering constants of materials [28].

E_1_/GPa	E_2_/GPa	E_3_/GPa	G_12_/GPa	G_13_/GPa	G_23_/GPa	ν_12_	ν_13_
54.3	54.3	3.3	3.26	2.17	2.17	0.04	0.01
ν_23_	X_T_/MPa	X_C_/MPa	Y_T_/MPa	Y_C_/MPa	S_12_/MPa	S_13_/MPa	S_23_/MPa
0.01	680	614.29	680	614.29	115.98	73.5	73.5

**Table 5:** Average tensile properties in three environments.

Static Properties	RTD	CTD	ETW
Maximum load/kN	16.85	19.02	10.75
Elastic modulus/GPa	12.10	13.63	11.07
Tensile strength/MPa	231.86	261.63	145.82

**Table 6:** Fatigue test results in three environments (stress ratio R = 0.0526).

Environments	Stress Level /%	Max Stress /MPa	Fatigue Life /Cycle
RTD	60	139.1	645,427, 77,795, 280,167, 925,905
	68	157.7	59,040, 15,576, 35,494, 33,051
	70	162.3	21,348, 15,576, 47,731, 39,773
	73	169.3	4213, 10,871, 11,142, 399,891
CTD	38	99.4	874,358, 1,207,366, 1,015,576, 840,721
	55	143.9	79,826, 88,424, 95,197, 82,243
	60	157	18,463, 29,169, 27,646, 20,312
	63	164.8	7866, 17,618, 9382, 20,316
ETW	45	65.6	1,124,138, 122,504, 917,306, 895,576
	50	72.9	213,125, 125,555, 123,153, 48,097
	56	81.7	70,968, 107,692, 35,724, 3890
	60	87.5	1608, 2988, 6730, 6038

**Table 7:** Comparison between the predicted values and the experimental values.

Stress Level	Life Cycle Number/Cycle			Logarithmic Life Number		
	Test	Simulation	Error	Simulation	Test	Error
RTD-60%	482,324	546,000	13.2%	5.53	5.74	3.8%
RTD-68%	35,790	36,500	2.0%	4.51	4.56	1.1%
RTD-70%	31,107	17,500	−43.7%	4.45	4.24	−4.72%
RTD-73%	16,500	6800	−58.8%	4.08	3.83	−6.1%
CTD-38%	984,505	1,014,000	3.0%	5.99	6.01	0.3%
CTD-55%	86,423	50,000	−42.1%	4.94	4.70	−4.9%
CTD-60%	23,898	27,000	13.0%	4.37	4.43	1.4%
CTD-63%	13,796	12,000	−13.0%	4.11	4.08	−0.7%
ETW-45%	764,881	780,000	2.0%	5.76	5.89	2.3%
ETW-50%	127,483	123,750	−3.0%	5.05	5.09	0.8%
ETW-56%	54,569	16,000	−70.7%	4.51	4.20	−6.9%
ETW-60%	4341	3400	−21.7%	3.57	3.53	−1.1%

## Data Availability

Data are contained within the article.
